# Pathogen distribution and risk factors for nosocomial infections in the operating room: A case-control study

**DOI:** 10.1097/MD.0000000000049971

**Published:** 2026-07-31

**Authors:** Hongsheng Yin, Weiqin Wu

**Affiliations:** a Department of Operating Room, The Second Affiliated Hospital of Zhejiang Chinese Medical University, Hangzhou City, Zhejiang Province, China.

**Keywords:** logistic regression, nosocomial infection, operating room, pathogen distribution, pneumonia, surgical site infection

## Abstract

Nosocomial infections remain a leading cause of postoperative morbidity and prolonged hospitalization, and they contribute to antimicrobial exposure and resistance selection. A retrospective case-control study was conducted among consecutive surgical patients treated in the operating room between January and December 2025; the sample comprised all eligible infected cases during the predefined study period and contemporaneous controls selected at a 1:1 ratio. Cases were patients who developed a postoperative nosocomial infection adjudicated using the Centers for Disease Control and Prevention and National Healthcare Safety Network definitions; controls were contemporaneous surgical patients without a nosocomial infection. Data were extracted from electronic clinical, anesthesia, operating room, laboratory, and microbiology systems. Group comparisons used Welch’s *t* test, Mann–Whitney *U* test, chi-square test, or Fisher’s exact test. Risk factors were assessed using univariate and multivariable logistic regression, with odds ratios and 95% confidence intervals. Culture positivity was identified in 44 of 56 cases (78.6%), with the highest specimen-level yield from operative-site and respiratory samples. Among 56 non-duplicate isolates, Gram-negative bacteria predominated (58.9%), followed by Gram-positive bacteria (32.1%) and fungi (8.9%); pathogen profiles differed by syndrome, with pneumonia largely caused by Gram-negative bacteria. In multivariable analysis, chronic obstructive pulmonary disease, longer preoperative length of stay, preoperative urinary catheterization, lower serum albumin, prolonged operative time, and intraoperative hypothermia were independently associated with infection risk. Operating room-associated nosocomial infections demonstrated syndrome-specific pathogen distributions and were linked to both host vulnerability and modifiable perioperative exposures, supporting integrated prevention and predictive strategies.

## 1. Introduction

Nosocomial infections are a major cause of postoperative morbidity. This study aimed to analyze the pathogen distribution and risk factors for nosocomial infections in surgical patients to inform prevention strategies. Nosocomial infections also prolong hospitalization, increase healthcare costs, and contribute to antimicrobial use and selection pressure that accelerate antimicrobial resistance. At the health-system level, infection prevention and control programs are increasingly evaluated against measurable outcomes, including surgical site infection (SSI) and procedure-associated pneumonia, with growing emphasis on implementation fidelity across perioperative workflows. The World Health Organization’s 2024 global report on infection prevention and control highlights persistent gaps in program implementation and underscores the downstream impact of healthcare-associated infections on patient safety and antimicrobial resistance.^[1,2]^ The operating room represents a high-consequence environment where infections can originate from multiple pathways, including inoculation at the incision, perioperative aspiration and airway colonization, device-related contamination, and hematogenous seeding. Contemporary prevention strategies emphasize bundled measures spanning the preoperative, intraoperative, and postoperative periods, integrating patient optimization, aseptic technique, environmental controls, and standardized perioperative processes. In 2023, the Society for Healthcare Epidemiology of America updated its implementation-focused guidance for preventing SSIs in acute-care hospitals, reinforcing the need for structured programs that prioritize high-yield practices and monitor adherence.^[3]^ However, the translation of such guidance into locally effective strategies requires granular knowledge of pathogen ecology and context-specific risk drivers.

Microbiological patterns are also evolving. Gram-negative organisms increasingly contribute to severe postoperative infections, including healthcare-associated pneumonia, and resistance phenotypes can complicate empiric therapy choices. Meanwhile, the environmental contribution to infection risk remains an area of active evaluation. Evidence syntheses have questioned whether laminar airflow provides consistent protection against SSIs in orthopedic operating rooms, suggesting that environmental investments should be aligned with demonstrable benefits and accompanied by attention to workflow factors that influence contamination risk.^[4,5]^ Similarly, intraoperative traffic and door-opening behaviors have been studied as modifiable exposure variables, reflecting interest in operational factors that may influence airborne microbial burden and aseptic discipline. Risk stratification for perioperative infection must also account for host vulnerability and procedural intensity. Prolonged operative duration has been associated with increased infection risk in procedure-specific analyses, supporting operative time as both a proxy for complexity and a potential target for process improvement.^[6,7]^ Nutritional and physiologic reserves, commonly indexed by serum albumin and frailty constructs, have been linked to adverse postoperative outcomes, including infectious complications, reinforcing the relevance of preoperative optimization.^[8]^ Device exposure is another relevant domain: early urinary catheter removal strategies have been evaluated in recent meta-analyses and randomized trials, reflecting ongoing efforts to reduce catheter-related infectious risk while maintaining functional recovery goals.^[9]^

Within this context, an integrated assessment of pathogen distribution across specimen sources and perioperative predictors can inform prevention priorities and support pragmatic, data-driven predictive strategies for operating room-associated nosocomial infections.

## 2. Methods

### 2.1. Study design

This study was approved by the Ethics Committee of The Second Affiliated Hospital of Zhejiang Chinese Medical University. This retrospective case-control study enrolled consecutive patients who underwent surgical procedures in the operating room at our institution between January 2025 and December 2025. Cases were defined as patients who developed a nosocomial infection temporally related to the index operation during hospitalization and/or within the standard postoperative surveillance window, with diagnoses adjudicated according to internationally accepted criteria (the Centers for Disease Control and Prevention [CDC]/National Healthcare Safety Network definitions for healthcare-associated infections and SSIs), while controls were patients undergoing surgery during the same period who did not meet the diagnostic criteria for a nosocomial infection. Controls were selected from the same source population and were comparable in terms of surgical specialty and calendar time. The study was conducted in accordance with the Declaration of Helsinki and was approved by the Institutional Medical Ethics Committee, and written informed consent was obtained from all participants or their legal representatives in accordance with institutional requirements.

### 2.2. Inclusion criteria and exclusion criteria

Inclusion criteria were as follows: receipt of operative treatment under sterile operating room conditions during the study period; availability of complete perioperative clinical records, including demographic data, comorbidities, anesthesia and operative details, antimicrobial exposure, microbiological cultures, and relevant laboratory/imaging results; and, for cases, microbiological and/or clinical evidence supporting the diagnosis of a nosocomial infection. Exclusion criteria were as follows: evidence of active infection before surgery or infection present/incubating at admission; procedures performed outside the main operating room environment (bedside or interventional suite procedures, if not within the predefined scope); readmissions for infection in which the index surgery was performed at another institution; severe missing data precluding outcome ascertainment or key covariate extraction; and perioperative antibiotic therapy initiated for a confirmed or suspected preoperative infection rather than for prophylaxis or extended prophylaxis.

### 2.3. Sample size justification

Sample size justification was performed post hoc because this retrospective case-control study included all eligible patients during the predefined study period. A total of 56 cases and 56 controls were included at a 1:1 ratio. Using a two-sided alpha level of 0.05 and 80% statistical power, we assessed whether the available sample size was adequate to detect clinically meaningful differences in major risk factors. For chronic obstructive pulmonary disease (COPD), one of the key clinically relevant binary exposures, the observed prevalence was 26.8% in cases and 7.1% in controls, corresponding to an odds ratio (OR) of 4.76 and Cohen’s *h* of 0.55. Based on a 2-independent-proportion comparison, approximately 53 participants per group would be required to achieve 80% power. Therefore, the inclusion of 56 participants per group in the present study provided adequate statistical power to detect this magnitude of difference. In addition, the observed between-group differences in operative time and serum albumin corresponded to moderate-to-large standardized effect sizes, further supporting the adequacy of the sample size for detecting major clinically relevant associations. Nevertheless, the study may still have been underpowered to detect small effects or associations involving uncommon exposures. Because this was a retrospective study, participants with incomplete data were excluded.

### 2.4. Data collection

Data were extracted from routinely maintained electronic sources, including the hospital information system and electronic medical records for demographics, admission and discharge dates, diagnoses, comorbidities, and medication administration records; the anesthesia information management system for American Society of Anesthesiologists (ASA) physical status classification, anesthesia technique, and intraoperative temperature recordings; the operating room management system for procedure category, surgical specialty, emergency status, incision and wound classification, operative start and end times, estimated blood loss, transfusion, and implant use; and the laboratory information system and microbiology database for perioperative laboratory tests and culture results. Comorbidities were defined using clinician-documented diagnoses and coded discharge diagnoses, supplemented by medication records when required for classification. The Charlson Comorbidity Index (CCI) was computed using established coding algorithms applied to diagnoses documented prior to the index operation. Preoperative length of stay was defined as the interval from hospital admission to the index operation. Preoperative antibiotic exposure was operationally defined as receipt of systemic antibiotics before incision beyond routine prophylaxis only when the indication was documented as prophylactic or risk-reduction related and no evidence supported treatment of an existing infection. To distinguish extended prophylaxis from therapy for an established infection, 2 investigators reviewed medication orders, administration timing, stated indications, perioperative notes, temperature and white blood cell count trends, preoperative culture results, imaging reports, and infectious disease or surgical consultation notes when available. Because patients with active infection before surgery or infection present/incubating at admission were excluded, antibiotics prescribed for confirmed or suspected preoperative infection were not counted as exposure; ambiguous cases were conservatively classified as treatment-related rather than extended prophylaxis and were excluded from the exposure definition. Preoperative device exposures were captured from procedural documentation, including urinary catheterization and central venous catheter placement recorded before or at the time of surgery. Laboratory variables were defined as the most recent preoperative measurements within 24 hours before surgery; if unavailable, the closest measurements within 72 hours was used. Intraoperative hypothermia was defined as any intraoperative core temperature below 36.0°C.

Microbiological specimens were obtained according to clinical indications. Specimen sources were categorized as wound or operative-site, respiratory, blood, or urine. Culture positivity was assessed at the patient level as at least 1 positive culture and at the specimen level by specimen type. For organism distribution analyses, isolates were deduplicated by retaining the first isolate per species per patient within the same infectious episode while preserving isolates from distinct specimen sources when clinically separate. Data quality was ensured through automated range and consistency checks and manual verification of a random subset of records against source documentation. All data were de-identified prior to analysis.

### 2.5. Statistical analysis

All analyses were performed using IBM SPSS Statistics, version 26.0 (IBM Corp., Armonk). Continuous variables were examined for distributional characteristics. Normally distributed variables were summarized as the mean ± standard deviation and compared using Welch’s independent-samples *t* test. Non-normally distributed variables were summarized as the median (interquartile range [IQR]) and compared using the Mann–Whitney *U* test. Categorical variables were reported as numbers (percentages) and compared using the chi-square test; Fisher’s exact test was used when expected cell counts were small. Culture positivity was calculated at the patient level and by specimen type, and specimen-level positivity across specimen categories was compared using the chi-square test. Pathogen distributions were summarized overall and by infection syndrome, and differences in major pathogen groups were assessed using the chi-square test or Fisher’s exact test, as appropriate. Risk factors for nosocomial infection were evaluated using logistic regression. Univariate logistic regression was first performed for each candidate variable, reporting ORs with 95% confidence intervals (CIs). Variables with univariate *P* < .10 and clinically important covariates were entered into multivariable logistic regression, with model parsimony guided by the number of outcome events. Multicollinearity was assessed using variance inflation factors. Adjusted ORs with 95% CIs were reported. All tests were two-sided, and *P* < .05 was considered statistically significant.

## 3. Results

### 3.1. Baseline and perioperative characteristics

Demographic characteristics were comparable between groups: mean age did not differ significantly (64.48 ± 8.29 vs 63.49 ± 7.67 years; *t* = 0.66, *P* = .510), and body mass index was similar (25.36 ± 3.21 vs 24.84 ± 3.15 kg/m^2^; *t* = 0.86, *P* = .394). The proportion of male patients was higher in the case group, although the difference was not statistically significant (64.3% vs 48.2%; χ^2^ = 2.96, *P* = .086). Regarding comorbidities and functional status, COPD was more frequent among cases than controls (26.8% vs 7.1%; χ^2^ = 7.67, *P* = .006). The CCI was also higher in cases (median 3.34 [IQR 2.14–4.21] vs 2.71 [IQR 1.71–3.55]; *U* = 1925.0, *P* = .038). No significant between-group differences were observed for diabetes mellitus, chronic kidney disease, malignancy, or immunosuppression (all *P* > .05). The distribution of ASA physical status classes was comparable between groups (χ^2^ = 3.70, *P* = .296).

For preoperative status and exposures, cases had a longer preoperative length of stay (median 4.52 [IQR 3.35–6.05] vs 3.57 [IQR 2.57–4.77] days; *U* = 2062.0, *P* = .004) and a higher rate of preoperative urinary catheterization (82.1% vs 60.7%; χ^2^ = 6.30, *P* = .012). Preoperative antibiotic exposure and preoperative central venous catheter placement did not differ significantly between groups (*P* = .122 and *P* = .281, respectively). Laboratory parameters showed lower serum albumin levels among cases (38.30 ± 4.25 vs 40.86 ± 3.44 g/L; *t* = −3.50, *P* < .001), whereas hemoglobin and white blood cell counts were similar (both *P* > .05). With respect to operative and anesthetic characteristics, cases had a longer operative duration (163.77 ± 53.80 vs 126.04 ± 47.39 minutes; *t* = 3.94, *P* < .001) and greater estimated blood loss (median 204.88 [IQR 154.28–318.64] vs 162.09 [IQR 124.16–224.74] mL; *U* = 1979.0, *P* = .017). Emergency surgery was more common in the case group (32.1% vs 16.1%; χ^2^ = 3.95, *P* = .047). Intraoperative hypothermia (<36°C) occurred more frequently among cases (32.1% vs 12.5%; χ^2^ = 6.23, *P* = .013). Surgical specialty distribution, wound class, transfusion, implant use, and anesthesia type did not differ significantly between groups (all *P* > .05; Table 1).

**Table 1:** Baseline and perioperative characteristics of cases and controls.

Variable	Case (n = 56)	Control (n = 56)	Test statistic	*P* value
Age, yr	64.48 ± 8.29	63.49 ± 7.67	*t* = 0.66	.510
BMI, kg/m^2^	25.36 ± 3.21	24.84 ± 3.15	*t* = 0.86	.394
Albumin, g/L	38.30 ± 4.25	40.86 ± 3.44	*t* = −3.50	<.001
Hemoglobin, g/L	125.87 ± 14.69	127.69 ± 13.26	*t* = −0.69	.492
WBC, ×10^9^/L	7.48 ± 2.42	7.52 ± 2.13	*t* = −0.10	.922
Operative time, min	163.77 ± 53.80	126.04 ± 47.39	*t* = 3.94	<.001
CCI	3.34 (2.14–4.21)	2.71 (1.71–3.55)	*U* = 1925.0	.038
Preoperative length of stay, d	4.52 (3.35–6.05)	3.57 (2.57–4.77)	*U* = 2062.0	.004
Estimated blood loss, mL	204.88 (154.28–318.64)	162.09 (124.16–224.74)	*U* = 1979.0	.017
Male sex, n (%)	36 (64.3)	27 (48.2)	χ^2^ = 2.96	.086
Diabetes mellitus, n (%)	23 (41.1)	18 (32.1)	χ^2^ = 0.96	.327
COPD, n (%)	15 (26.8)	4 (7.1)	χ^2^ = 7.67	.006
CKD, n (%)	9 (16.1)	5 (8.9)	χ^2^ = 0.37	.541
Malignancy, n (%)	16 (28.6)	14 (25.0)	χ^2^ = 0.40	.526
Immunosuppression, n (%)	6 (10.7)	2 (3.6)	Fisher’s exact	.679
Preoperative antibiotic exposure, n (%)	17 (30.4)	10 (17.9)	χ^2^ = 2.39	.122
Preoperative urinary catheterization, n (%)	46 (82.1)	34 (60.7)	χ^2^ = 6.30	.012
Preoperative CVC, n (%)	17 (30.4)	12 (21.4)	χ^2^ = 1.16	.281
Emergency surgery, n (%)	18 (32.1)	9 (16.1)	χ^2^ = 3.95	.047
Intraoperative blood transfusion, n (%)	14 (25.0)	9 (16.1)	χ^2^ = 0.16	.686
Implant use, n (%)	18 (32.1)	17 (30.4)	χ^2^ = 0.16	.686
General anesthesia, n (%)	48 (85.7)	46 (82.1)	χ^2^ = 0.26	.607
Intraoperative hypothermia (<36°C), n (%)	18 (32.1)	7 (12.5)	χ^2^ = 6.23	.013
ASA physical status (I/II/III/IV), n (%)	3 (5.4)/21 (37.5)/26 (46.4)/6 (10.7)	8 (14.3)/23 (41.1)/22 (39.3)/3 (5.4)	χ^2^ = 3.70	.296
Wound class (I/II/III/IV), n (%)	18 (32.1)/25 (44.6)/10 (17.9)/3 (5.4)	28 (50.0)/16 (28.6)/9 (16.1)/3 (5.4)	χ^2^ = 4.20	.240
Surgical specialty (Gen/Ortho/Neuro/Gyn/Urol), n (%)	18 (32.1)/11 (19.6)/10 (17.9)/11 (19.6)/6 (10.7)	20 (35.7)/7 (12.5)/8 (14.3)/12 (21.4)/9 (16.1)	χ^2^ = 1.86	.762

ASA = American Society of Anesthesiologists, BMI = body mass index, CCI = Charlson Comorbidity Index, CKD = chronic kidney disease, COPD = chronic obstructive pulmonary disease, CVC = central venous catheter, Gen = general surgery, Gyn = gynecology, Neuro = neurosurgery, Ortho = orthopedics, Urol = urology, WBC = white blood cell count.

### 3.2. Pathogen distribution and specimen sources

Microbiological specimens were obtained according to clinical indications. At the patient level, at least 1 culture was positive in 44/56 cases (78.6%). At the specimen level, wound/operative-site samples and respiratory samples showed the highest positivity (81.3% and 81.8%, respectively), whereas blood and urine cultures yielded lower positivity (50.0% and 60.0%, respectively). When specimen-level positivity was compared across specimen types, the overall difference did not reach statistical significance (χ^2^ = 6.60, *P* = .086; Table 2).

**Table 2:** Specimen sources and culture positivity.

Specimen type	Specimens collected, n	Culture-positive, n (%)	Test statistic	*P* value
Wound/operative-site	32	26 (81.3)		
Respiratory (sputum/aspirate)	22	18 (81.8)		
Blood	14	7 (50.0)		
Urine	10	6 (60.0)		
Overall (specimen-level comparison)	78	57 (73.1)	χ^2^ = 6.60	.086
Patient-level positivity	56	44 (78.6)	–	–

Across all non-duplicate isolates (n = 56, derived from 44 culture-positive patients), Gram-negative bacteria accounted for 33/56 (58.9%), Gram-positive bacteria for 18/56 (32.1%), and fungi for 5/56 (8.9%). The most frequently identified organisms were *Escherichia coli* (21.4%), *Klebsiella pneumoniae* (16.1%), *Staphylococcus aureus* (12.5%), *Pseudomonas aeruginosa* (10.7%), and *Acinetobacter baumannii* (10.7%). Pathogen profiles differed by infection syndrome: SSI isolates were enriched for Gram-positive organisms, whereas pneumonia isolates were predominantly Gram-negative. Using isolate-level categorization, Gram-negative organisms represented 12/28 (42.9%) isolates in SSI versus 19/20 (95.0%) in pneumonia (χ^2^ = 11.68, *P* < .001). Culture positivity rates were comparable between SSI and pneumonia (80.0% vs 83.3%; Fisher’s exact *P* = 1.000; Table 3). Regarding infection complexity, 33/44 (75.0%) culture-positive cases were monomicrobial, and 11/44 (25.0%) were polymicrobial (10 dual-pathogen infections and 1 triple-pathogen infection), yielding 56 total isolates. The proportion of polymicrobial infections was similar between culture-positive SSI and pneumonia cases (25.0% vs 26.7%; Fisher’s exact *P* = 1.000).

**Table 3:** Pathogen distribution overall and by infection syndrome.

Organism	Overall isolates (n = 56), n (%)	SSI isolates (n = 28), n (%)	Pneumonia isolates (n = 20), n (%)
Gram-negative bacteria	33 (58.9)	12 (42.9)	19 (95.0)
*Escherichia coli*	12 (21.4)	8 (28.6)	3 (15.0)
*Klebsiella pneumoniae*	9 (16.1)	3 (10.7)	6 (30.0)
*Pseudomonas aeruginosa*	6 (10.7)	1 (3.6)	5 (25.0)
*Acinetobacter baumannii*	6 (10.7)	0 (0.0)	5 (25.0)
Gram-positive bacteria	18 (32.1)	14 (50.0)	1 (5.0)
*Staphylococcus aureus*	7 (12.5)	6 (21.4)	1 (5.0)
Coagulase-negative *Staphylococcus* spp.	4 (7.1)	3 (10.7)	0 (0.0)
*Enterococcus faecalis*	4 (7.1)	3 (10.7)	0 (0.0)
*Enterococcus faecium*	3 (5.4)	2 (7.1)	0 (0.0)
Fungi	5 (8.9)	2 (7.1)	0 (0.0)
*Candida albicans*	3 (5.4)	1 (3.6)	0 (0.0)
*Candida glabrata*	2 (3.6)	1 (3.6)	0 (0.0)

Isolate-level comparison (SSI vs pneumonia): Gram-negative vs non–Gram-negative isolates: 12/28 versus 19/20; χ^2^ = 11.68, *P* < .001.

Patient-level culture positivity (SSI vs pneumonia): 24/30 versus 15/18; Fisher’s exact *P* = 1.000.

### 3.3. Univariate analysis

Univariate logistic regression was performed to evaluate the association between each candidate predictor and nosocomial infection. Continuous variables were retained as continuous predictors to preserve information and were scaled for interpretability (age per 10 years, albumin per 5 g/L, hemoglobin per 10 g/L, operative time per 30 minutes, estimated blood loss per 100 mL, and preoperative length of stay per day). For multivariable model candidacy, variables with *P* < .10 in univariate analysis were prespecified to enter the multivariable model; additionally, variables considered clinically important (e.g., perioperative antibiotic exposure and estimated blood loss) could be forced into the multivariable model even if *P* ≥ .10. In univariate models, COPD (OR: 4.76, *P* = .009), higher CCI (OR: 1.32 per 1-point increase, *P* = .036), longer preoperative length of stay (OR: 1.30 per day, *P* = .014), preoperative urinary catheterization (OR: 2.98, *P* = .014), lower albumin (OR: 0.45 per 5 g/L increase, *P* = .006), longer operative time (OR: 2.09 per 30 minutes, *P* < .001), and intraoperative hypothermia (OR: 3.32, *P* = .015) were significantly associated with infection risk. Emergency surgery showed a borderline association (OR: 2.47, *P* = .050), and male sex met the screening threshold (OR: 1.93, *P* = .088). Other covariates, including diabetes mellitus, chronic kidney disease, malignancy, ASA III to IV, wound class III to IV, transfusion, implant use, and general anesthesia, were not significantly associated with infection in univariate analysis (all *P* > .05; Table 4).

**Table 4:** Univariate logistic regression for predictors of nosocomial infection.

Variable	OR (95% CI)	*P* value
Age (per 10 yr)	0.99 (0.62–1.56)	.954
Male sex	1.93 (0.91–4.12)	.088
BMI (per 1 kg/m^2^)	1.05 (0.93–1.19)	.416
Diabetes mellitus	1.47 (0.68–3.19)	.328
COPD	4.76 (1.47–15.42)	.009
CKD	1.95 (0.61–6.25)	.259
Malignancy	1.20 (0.52–2.77)	.670
Immunosuppression	3.24 (0.62–16.80)	.162
ASA III–IV	1.65 (0.78–3.49)	.187
CCI (per 1 point)	1.32 (1.02–1.71)	.036
Preoperative length of stay (per d)	1.30 (1.05–1.59)	.014
Preoperative antibiotic exposure	2.01 (0.82–4.88)	.126
Preoperative urinary catheterization	2.98 (1.25–7.10)	.014
Preoperative CVC	1.60 (0.68–3.76)	.283
Albumin (per 5 g/L)	0.45 (0.26–0.80)	.006
Hemoglobin (per 10 g/L)	0.85 (0.66–1.11)	.233
Operative time (per 30 min)	2.09 (1.52–2.87)	<.001
Estimated blood loss (per 100 mL)	1.47 (0.91–2.38)	.113
Emergency surgery	2.47 (1.00–6.13)	.050
Wound class III–IV	1.11 (0.46–2.70)	.821
Intraoperative blood transfusion	1.74 (0.68–4.43)	.245
Implant use	1.09 (0.49–2.42)	.838
General anesthesia	1.30 (0.47–3.59)	.607
Intraoperative hypothermia (<36°C)	3.32 (1.26–8.75)	.015

ASA = American Society of Anesthesiologists, BMI = body mass index, CCI = Charlson Comorbidity Index, CI = confidence interval, CKD = chronic kidney disease, COPD = chronic obstructive pulmonary disease, CVC = central venous catheter, OR = odds ratio.

### 3.4. Multivariable analysis

Candidate variables were selected a priori based on univariate screening (*P* < .10) and clinical relevance while maintaining an appropriate events-per-variable ratio given the 56 outcome events. To reduce multicollinearity, clinically overlapping variables were reviewed, and collinearity diagnostics indicated acceptable inflation (all variance inflation factors < 2.5). The final model included COPD, CCI, preoperative length of stay (per day), preoperative urinary catheterization, serum albumin (per 5 g/L), operative time (per 30 minutes), and intraoperative hypothermia (<36°C). Emergency surgery and male sex were not retained in the final model after accounting for confounding and model parsimony.

In the adjusted analysis, COPD remained independently associated with higher odds of nosocomial infection (adjusted OR [aOR]: 3.61, 95% CI: 1.05–12.43; *P* = .042). Longer preoperative length of stay was associated with increased infection risk (aOR: 1.22 per day, 95% CI: 1.05–1.41; *P* = .009), and preoperative urinary catheterization was also independently associated with infection (aOR: 2.39, 95% CI: 1.01–5.66; *P* = .047). Higher serum albumin was protective (aOR: 0.57 per 5 g/L increase, 95% CI: 0.35–0.92; *P* = .021). Operative time remained a strong independent predictor (aOR: 1.74 per 30 minutes, 95% CI: 1.26–2.41; *P* < .001), and intraoperative hypothermia was associated with increased odds of infection (aOR: 2.68, 95% CI: 1.03–6.94; *P* = .043). CCI demonstrated a borderline independent association (aOR: 1.21 per 1-point increase, 95% CI: 0.99–1.47; *P* = .061; Table 5). The final model was interpreted as an explanatory multivariable model for adjusted associations; formal discrimination and calibration indices were not generated in the present retrospective analysis.

**Table 5:** Multivariable logistic regression identifying independent predictors of nosocomial infection.

Variable	Adjusted OR (95% CI)	*P* value
COPD (yes vs no)	3.61 (1.05–12.43)	.042
CCI (per 1 point)	1.21 (0.99–1.47)	.061
Preoperative length of stay (per d)	1.22 (1.05–1.41)	.009
Preoperative urinary catheterization (yes vs no)	2.39 (1.01–5.66)	.047
Albumin (per 5 g/L increase)	0.57 (0.35–0.92)	.021
Operative time (per 30 min)	1.74 (1.26–2.41)	<.001
Intraoperative hypothermia (<36°C) (yes vs no)	2.68 (1.03–6.94)	.043

CCI = Charlson Comorbidity Index, CI = confidence interval, COPD = chronic obstructive pulmonary disease, OR = odds ratio.

## 4. Discussion

This study integrated microbiological profiling with perioperative risk factor assessment to clarify the determinants of nosocomial infections among surgical patients managed in the operating room context. Several findings warrant emphasis. First, culture positivity was high at the patient level, and operative-site and respiratory specimens yielded the highest positivity. Second, Gram-negative organisms predominated overall, with marked syndrome-specific differences: pneumonia isolates were overwhelmingly Gram-negative, whereas SSI isolates showed a comparatively higher proportion of Gram-positive organisms. Third, after adjustment, COPD, longer preoperative length of stay, preoperative urinary catheterization, lower serum albumin, longer operative time, and intraoperative hypothermia remained independently associated with nosocomial infection. Collectively, these observations support a prevention approach focused on host vulnerability, preoperative exposure intensity, and modifiable intraoperative processes.

The predominance of Gram-negative organisms in this cohort is consistent with contemporary epidemiology of hospital-acquired infections and the recognized role of Gram-negative bacteria in healthcare-associated pneumonia, particularly in critically ill or postanesthesia patients. Recent reviews of hospital-acquired and ventilator-associated pneumonia continue to describe Gram-negative bacteria as frequent pathogens and a major driver of therapeutic complexity.^[10,11]^ The observed syndrome-specific distribution is clinically coherent. Postoperative pneumoniacommonly arises from aspiration- and colonization-related mechanisms in the aerodigestive tract, which tend to favor Gram-negative flora, whereas SSIs remain more closely linked to skin flora and operative-site contamination pathways. Although the present study did not include susceptibility profiling, the organism spectrum underscores the importance of aligning empiric antimicrobial decisions with local microbiology and the suspected syndrome, followed by de-escalation based on culture results to support antimicrobial stewardship. Specimen-level findings further support syndrome-directed sampling. Higher positivity in wound/operative-site and respiratory specimens is expected because these samples are typically obtained when the clinical likelihood of infection is higher (e.g., purulent drainage or radiographic infiltrates), while blood culture positivity may be lower because of intermittent bacteremia and prior antibiotic exposure. Standardized criteria for specimen collection and timing may help balance diagnostic yield with the avoidance of unnecessary culturing, which can generate false-positive results or promote inappropriate antibiotic escalation.

COPD remained an independent predictor of nosocomial infection after adjustment. COPD is associated with impaired mucociliary clearance, baseline airway inflammation, altered respiratory microbiota, and reduced pulmonary reserve, factors that can increase susceptibility to postoperative pneumonia and impair recovery from perioperative physiologic stress. Contemporary perioperative pulmonary optimization discussions continue to identify COPD as a central determinant of postoperative pulmonary complications and emphasize targeted risk assessment and optimization strategies.^[12,13]^ While the CCI showed only borderline statistical significance in the final model, the directionality was consistent with an incremental risk associated with increasing comorbidity burden. This pattern suggests that multimorbidity may contribute through overlapping mechanisms (functional limitation, frailty, inflammatory burden) that are partially captured by more proximate predictors such as albumin level and length of preoperative hospitalization.

Longer preoperative length of stay was independently associated with infection. Preoperative hospitalization may increase exposure to healthcare-associated flora, broaden opportunities for invasive procedures, and intensify antimicrobial selection pressures, all of which can facilitate colonization and subsequent infection. Evidence linking prolonged preoperative hospitalization to infection risk has been reported in recent observational research, including analyses specifically evaluating preoperative stay and SSI risk.^[14,15]^ From a systems perspective, these findings support efforts to streamline preoperative work-up, reduce avoidable inpatient waiting time, and shift optimization activities to outpatient pathways where feasible. Such approaches should be implemented cautiously, ensuring that shortened preoperative stays do not compromise clinical stability or preoperative preparation. Preoperative urinary catheterization was also independently associated with infection. Urinary catheters are well-established risk factors for catheter-associated urinary tract infection, and contemporary CDC recommendations emphasize minimizing catheter use and duration, with insertion only for appropriate indications and prompt removal when no longer needed. Beyond catheter-associatedurinary tract infection, perioperative catheter carriage may correlate with broader infectious risk through several pathways, including facilitation of microbial entry, increased manipulation during perioperative care, and confounding by illness severity or immobility. Importantly, recent perioperative evidence has linked perioperative urinary catheter use to Gram-negative SSI in surgical populations, supporting biological plausibility for catheter-associatedcontributions beyond urinary tract infection alone.^[16]^ These observations justify integrating device stewardship into operating room infection prevention strategies, including strict indication criteria, aseptic insertion and maintenance bundles, and removal protocols embedded in perioperative checklists.

Higher serum albumin was protective, consistent with the extensive literature linking hypoalbuminemia to postoperative infectious complications. Albumin reflects nutritional reserves and systemic inflammatory activity; low albumin may correspond to impaired wound healing, altered immune responses, and increased susceptibility to infection. Recent systematic evaluations focusing on nutritional status and SSI outcomes in surgical populations continue to identify nutritional vulnerability as a relevant determinant of infection risk.^[17,18]^ Clinically, this supports routine preoperative nutritional screening and targeted interventions (dietary support, correction of protein-energy malnutrition, and consideration of broader frailty assessment) in high-risk patients, especially when prolonged operative time is anticipated or when preoperative hospitalization is unavoidable. Operative time remained a strong independent predictor. Prolonged procedures may increase the exposure of tissues to environmental contaminants, prolong periods of reduced perfusion and physiologic stress, and reflect technical complexity. Recent clinical analyses continue to report prolonged operative duration as a meaningful contributor to SSI risk in abdominal and other surgeries.^[19]^ While operative time is partly non-modifiable (case complexity), improvement opportunities exist in workflow efficiency, preoperative planning, team coordination, and reduction of avoidable delays. Intraoperative hypothermia was independently associated with infection. Hypothermia may impair immune function, reduce tissue oxygenation, and influence coagulation and wound healing. Recent meta-analyses have evaluated this association, with some reporting increased SSI risk under certain conditions or temperature thresholds while acknowledging heterogeneity across study designs and populations.^[20]^ Regardless of variability in effect size, normothermia remains a core component of SSI prevention bundles and is routinely emphasized in guidance documents and quality improvement frameworks. Operationally, these findings support implementing active warming strategies (including prewarming when appropriate), continuous temperature monitoring, and prompt correction of hypothermia – particularly for long procedures and patients with baseline vulnerability.

The adjusted risk factor profile in this study maps onto 3 actionable domains: host-focused optimization, identification and optimization of COPD and nutritional vulnerability (albumin) before surgery; exposure reduction, minimizing preoperative inpatient days and device use, particularly urinary catheterization, consistent with CDC recommendations; and intraoperative process control, maintaining normothermia and improving operative efficiency. Together, these strategies align with bundled prevention approaches promoted in contemporary SSI prevention guidance and toolkits. The organism spectrum further indicates that empiric management for suspected postoperative pneumonia may require attention to Gram-negative coverage tailored to local susceptibility patterns, while SSI empiric choices may be more dependent on incision class, local *S aureus* prevalence, and procedure type, pending culture results.^[21,22]^

Interpretation should consider several limitations. The case-control design is susceptible to selection bias and residual confounding. Cultures were obtained based on clinical indication rather than standardized prospective protocols, which can influence positivity rates and organism distribution. The sample size, while adequate for a parsimonious multivariable model, limited precision for uncommon exposures and constrained the number of covariates that could be included without overfitting. Susceptibility patterns and resistance phenotypes were not incorporated, limiting inference about empiric antibiotic selection beyond organism distribution. In addition, this was a single-center study; therefore, pathogen distribution and risk factorsmay reflect regional microbiological ecology, institutional case mix, perioperative workflows, and local infection-control practices. The findings should, therefore, be extrapolated cautiously to other settings, although they provide a practical reference for multicenter validation and for the development of localized prevention strategies. Finally, without molecular typing, transmission pathways (endogenous colonization vs cross-transmission) cannot be determined. Although the sample size was considered adequate for a parsimonious explanatory multivariable model, it limited precision for uncommon exposures and constrained the number of covariates that could be included without overfitting. Formal calibration and discrimination measures were not calculated; therefore, the multivariable model should be interpreted as identifying adjusted associations rather than serving as a validated prediction model.

## 5. Conclusion

This study found syndrome-specific pathogen distributions with an overall Gram-negative predominance and identified independent perioperative predictors of nosocomial infection, including COPD, prolonged preoperative hospitalization, urinary catheterization, hypoalbuminemia, longer operative time, and intraoperative hypothermia. These findings support prevention strategies emphasizing risk stratification, nutritional and pulmonary optimization, device stewardship, reduction of preoperative exposure time when clinically feasible, maintenance of normothermia, and improved operative efficiency, alongside culture-guided antimicrobial management informed by local epidemiology. Given the single-center design, extrapolation should be cautious, but the results provide a useful reference for multicenter studies and for localized, bundle-based perioperative prevention strategies.

## Author contributions

**Conceptualization:** Hongsheng Yin, Weiqin Wu.

**Data curation:** Hongsheng Yin, Weiqin Wu.

**Formal analysis:** Hongsheng Yin, Weiqin Wu.

**Funding acquisition:** Hongsheng Yin.

**Investigation:** Hongsheng Yin.

**Writing – original draft:** Hongsheng Yin.

**Writing – review & editing:** Hongsheng Yin.
